# GATAD2B is required for pre‐implantation embryonic development by regulating zygotic genome activation

**DOI:** 10.1111/cpr.13647

**Published:** 2024-04-12

**Authors:** Yuling Lin, Lina Yu, Qian Xu, Panpan Qiu, Yang Zhang, Xiaohan Dong, Guijun Yan, Haixiang Sun, Guangyi Cao

**Affiliations:** ^1^ Center for Reproductive Medicine and Obstetrics and Gynecology, Nanjing Drum Tower Hospital Clinical College of Nanjing Medical University Nanjing China; ^2^ State Key Laboratory of Reproductive Medicine and Offspring Health, Nanjing Medical University Nanjing China; ^3^ Center for Reproductive Medicine and Obstetrics and Gynecology, Nanjing Drum Tower Hospital, Affiliated Hospital of Medical School, Nanjing University Nanjing China; ^4^ Key Laboratory of Reproductive Medicine of Guangdong Province Guangzhou China

## Abstract

Major zygotic genome activation (ZGA) occurs at the late 2‐cell stage and involves the activation of thousands of genes, supporting early embryonic development. The reasons underlying the regulation of ZGA are not clear. Acetylation modifications of histone tails promote transcriptional activation, and the maternal deletion of H4K16ac leads to failure in ZGA. GATAD2B is one of the core subunits of the nucleosome remodelling and histone deacetylation (NuRD) complex. Our research has shown that GATAD2B exhibits specific nucleus localization and high protein expression from the late 2‐cell stage to the 8‐cell stage. This intriguing phenomenon prompted us to investigate the relationship between GATAD2B and the ZGA. We discovered a distinctive pattern of GATAD2B, starting from the late 2‐cell stage with nuclear localization. GATAD2B depletion resulted in defective embryonic development, including increased DNA damage at morula, decreased blastocyst formation rate, and abnormal differentiation of ICM/TE lineages. Consistent with the delay during the cleavage stage, the transcriptome analysis of the 2‐cell embryo revealed inhibition of the cell cycle G2/M phase transition pathway. Furthermore, the GATAD2B proteomic data provided clear evidence of a certain association between GATAD2B and molecules involved in the cell cycle pathway. As hypothesized, GATAD2B‐deficient 2‐cell embryos exhibited abnormalities in ZGA during the maternal‐to‐embryonic transition, with lower expression of the major ZGA marker MERVL. Overall, our results demonstrate that GATAD2B is essential for early embryonic development, in part through facilitating ZGA.

## Graphical abstract

Major ZGA occurs at the late‐2 cell stage and involves the activation of thousands of genes, supporting early embryonic development. GATAD2B is one of the core subunits of the nucleosome remodelling and histone deacetylation (NuRD) complex. Our results demonstrate that GATAD2B is essential for pre‐implantation embryonic development, in part through facilitating ZGA.

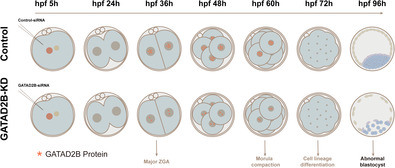

## 1 INTRODUCTION

After fertilization, the zygote of mammals undergoes reprogramming from highly differentiated gametes to totipotent two‐cell stage, forming a pluripotent blastocyst through cleavage. During the transition from oocyte to embryo, the embryo goes through several stages, including the degradation of maternal mRNA and the zygotic genome activation (ZGA).^1^ The activation of the embryonic genome involves two waves, minor ZGA and major ZGA.^2^, ^3^ Minor ZGA primarily occurs at the mid‐one‐cell stage, accompanied by the activation of a handful of genes in mice. Subsequently, in late two‐cell (L2C) embryos, thousands of genes are activated, known as major ZGA.^2^, ^4^

In recent years, there have been some new advances in the activation and regulation of ZGA. During the process of embryonic genome activation, RNA polymerase II (RNA Pol II) is an important transcription machinery that undergoes loading, pre‐configuration, and elongation for gene expression.^5^ Knockout of maternal TDP‐43 in mouse embryos resulted in the loss of RNA Pol II foci and arrest at the two‐cell stage.^6^ Inhibiting the activity of minor ZGA using a transcription elongation inhibitor DRB leads to the arrest of mouse embryos at the two‐cell stage.^4^ Knockdown or chemical inhibition of *Nr5a2* results in abnormal activation of 70% of ZGA genes and subsequently reduces the blastocyst formation rate.^7^ Impaired RNA Pol II pre‐configuration in *Obox* mutants in a genetic knockout model was accompanied by defective ZGA and chromatin accessibility transition.^8^ Although these findings have provided insight into certain mechanisms of ZGA regulation, numerous mysteries pertaining to this vital process of life initiation remain unresolved.

The acetylation modification of histone tails is known to promote transcriptional activation.^9^ Among the histone modifications associated with transcriptional activation regulation, H4K16ac is consistently highly expressed in mouse oocytes and early embryos. Maternal deletion of H4K16ac leads to abnormal recruitment of RNA Pol II, defects in the 3D assembly of active genomic compartments, and subsequently lower activation of ZGA gene expression.^10^ H4K16ac can be regulated by the nucleosome remodelling and histone deacetylation (NuRD) complex through the coordinated involvement of the deacetylase and the ZMYND8 subunit.^11^ The NuRD complex is a 1 MDa multi‐subunit protein complex consisting of various subunits, including *Hdac1*/*2*, *Mta1*/*2*, *Rbbp4*/*7*, *Chd3*/*4*, and *Gatad2a*/*2b*.^12^, ^13^ *Gatad2b*, also known as p66b, recruits *Mbd2*, another component of the NuRD complex, to DNA and histones, thereby affecting histone deacetylation and transcriptional repression.^14^ An opposing regulation of *Gatad2b* by *Mkk6* phosphorylation increases histone acetylation levels and enhances pluripotency gene expression.^15^ Recently, it has been reported that *Gatad2b* is sumoylated to enhance the formation of NuRD complexes.^16^, ^17^, ^18^ Additionally, *Gatad2b* can directly interact with histone acetylation sites, including H3K9ac, H3K27ac, and H4K16ac.^15^ Although GATAD2B plays a crucial role in somatic cells, its function in early embryonic development remains unclear.

In this study, we observed a unique phenomenon of GATAD2B shuttling between the cytoplasm and nucleus during embryonic development. Depletion of GATAD2B results in subsequent abnormalities in early embryonic development, including decreased blastocyst formation rate, increased DNA damage, and abnormal differentiation of the inner cell mass (ICM) and trophectoderm (TE) lineages during the blastocyst stage. Consistent with the slow development of the 4‐/8‐cell stage, the transcriptome of the 2‐cell stage suggests inhibition of the cell cycle G2/M phase transition pathway. Importantly, we found abnormal ZGA in GATAD2B‐deficient two‐cell embryos during the maternal‐to‐embryo transition process.

## 2 MATERIALS AND METHODS

### 2.1 Animals

All animal experiments were conducted in accordance with the guidelines of the Institutional Animal Care and Use Committee of Nanjing Drum Tower Hospital (SYXK 2021–0509). ICR mice were obtained from Beijing Vital River Laboratory Animal Technology. The mice were provided with regular chow and housed in a controlled environment with a 12:12‐h light–dark cycle at 22°C.

### 2.2 Embryo Collection and Culture

Embryo collection and culture for in vitro fertilization experiments were performed using 8‐week‐old female B6D2F1 mice and 12‐week‐old male B6D2F1 mice. The 8‐week‐old female B6D2F1 mice were injected with 10 IU of pregnant mare serum gonadotropin (PMSG; Ningbo Sansheng Biological Technology, 110,914,564), followed by an injection of 10 IU of human chorionic gonadotropin (hCG; Ningbo Sansheng Biological Technology, 110,911,282) 48 hours later for superovulation. Fifteen hours after hCG injection, MII oocytes were collected from the ampulla of the fallopian tubes and transferred to G‐IVF™ PLUS (Vitrolife, 10,136). The sperm from male B6D2F1 mice was capacitated in G‐IVF™ PLUS for 1 h, and then, the MII oocytes from female B6D2F1 mice were fertilized with the caudal epididymal sperm from male B6D2F1 mice in G‐IVF™ PLUS. The fertilized oocytes were washed 4–6 h after fertilization and cultured in G‐1™ PLUS supplemented with HSA (Vitrolife, 10,128, equilibrated overnight at 37°C in 5% CO_2_ before use). Embryos at 8, 24, 36, 48, 60, 72, and 84 h post‐fertilization were collected at the zygote, early 2‐cell, late 2‐cell, 4‐cell, 8‐cell, morula, and blastocyst stages, respectively.

### 2.3 Antibodies

The antibodies used in this study are listed in Table S1.

### 2.4 Microinjection

The mouse embryos obtained from in vitro fertilization were microinjected at the zygote stage using a FemtoJet microinjector (Eppendorf) with approximately 10 pL of targeted *Gatad2b* small interfering RNA (siRNA) (25 μM). The siRNA sequence for *Gatad2b* is as follows: 5′‐GCAGCCAAUAGCGAGUUUATT‐3′, 5′‐GGGACAACAAGGCUUAUCUTT‐3′, 5′‐CCCGAUCCAUGCUUUCAAATT‐3′. After injection, the embryos were transferred to G‐1™ PLUS culture medium (Vitrolife, 10,128) that had been equilibrated overnight at 37°C in a culture chamber (5% O_2_, 5% CO_2_, and 90% N_2_) and continuously observed.

### 2.5 Immunofluorescence

Embryo samples were fixed in PBS containing 4% paraformaldehyde (PFA) at room temperature for 30 min. The samples were permeabilized with 0.5% Triton X‐100 at room temperature for 20 min, followed by blocking with PBS containing 5% FBS at room temperature for 1 h. Primary antibodies were incubated overnight at 4°C, followed by three washes with PBST (PBS containing 0.1% Triton X‐100) for 5 min each. The samples were then incubated with specific fluorescently labelled secondary antibodies at room temperature for 1 h (all fluorescent secondary antibodies were from Thermo Fisher). After three washes with PBST, the embryos were placed on glass slides and mounted with ProLong™ Gold Antifade Mountant with DAPI (Invitrogen™, Cat no: P36931). Imaging was performed using a Zeiss LSM 780 laser scanning confocal microscope.

### 2.6 Western Blotting

Mouse embryo samples were collected with 30 embryos per time point, lysed in sample buffer, and boiled for 5 min at 100°C before loading for Western blot analysis. Denatured proteins were separated by 10% sodium dodecyl sulphate–polyacrylamide gel electrophoresis (SDS‐PAGE) and transferred onto PVDF membranes. After blocking with PBS containing 5% skim milk at room temperature for 1 h, the membranes were incubated overnight at 4°C with the following primary antibodies: GATAD2B rabbit polyclonal antibody (1:1000; 25,679‐1‐AP, Proteintech) and mouse anti‐β‐actin antibody (1:1000; AF0003, Beyotime). After three washes with TBS‐T, the membranes were incubated with horseradish peroxidase‐conjugated secondary antibodies in blocking buffer for 1 h at room temperature. Following three washes with TBS‐T, protein bands were visualized using the ECL Plus Western Blotting Detection System (GE Healthcare).

### 2.7 Quantitative RT‐PCR analyses

cDNA from embryos was obtained using a Single Cell Sequence Specific Amplification Kit (P621; Vazyme) according to the manufacturer's instructions. Briefly, amplification primers for different target genes were mixed to create a primer pool (final concentration of each primer: 0.1 μM). The reaction system containing cell samples and the primer pool was prepared in nuclease‐free centrifuge tubes and reverse transcribed for 15 cycles as recommended in the protocol. The resulting cDNA was used for subsequent differential gene expression analysis. To minimize experimental variation between groups, at least 10 embryos were used in each sample group. The diluted cDNA (100‐fold dilution) was subjected to fluorescent quantitative PCR using 2 μL of the diluted cDNA on a LightCycler 480 II system (Roche). The expression levels of genes were determined using the 2^−ΔΔCt^ method. The primers used in qPCR are listed in Table S2.

### 2.8 RNA‐seq

Embryos injected during the pronuclear stage were cultured in G‐1™ PLUS embryo culture medium. Developmental observations were made, and at the same stage, three groups of samples were collected. The embryos were lysed using a lysis buffer containing RNA inhibitors. Each group consisted of 5 control late 2‐cell embryos and 5 GATAD2B‐KD late 2‐cell embryos for RNA‐seq analysis. The full‐length cDNA amplification products were generated using the Single Cell Full‐Length mRNA Amplification Kit (N712; Vazyme). Subsequently, the TruePrep® DNA Library Prep Kit V2 for Illumina (TD503; Vazyme) was used according to the manufacturer's instructions. The libraries were sequenced on the Illumina HiSeq X Ten platform. Differential gene expression analysis and normalization were performed using the DESeq2 package (v1.28.1). Differential expression transcripts were subjected to Gene Set Enrichment Analysis using the DAVID (https://david.ncifcrf.gov/) and Metascape (https://metascape.org/) platforms.

### 2.9 Immunoprecipitation combined with mass spectrometry identification

Ovaries were collected from 3‐week‐old female ICR mice at 44–46 h after PMSG injection. Ten ovaries were pooled as one group, and immunoprecipitation (IP) of GATAD2B was performed on the samples after lysis. Briefly, GATAD2B antibody (Cat no: 25679‐1‐AP; Proteintech) was incubated with ovarian lysate, followed by the addition of magnetic beads to capture the antibody–antigen complex. Co‐precipitated proteins were then analysed by mass spectrometry (Thermo ScientificTM Q ExactiveTM HFX). The mass spectrometry data were analysed using Proteome Discoverer 2.4, and the identified interacting proteins are listed in Table S3.

### 2.10 Statistical analysis

Unless otherwise indicated, data are presented as mean ± SEM. Differences between the two groups were evaluated using Student's *t*‐test. Multiple comparisons among more than two groups were analysed by one‐way ANOVA followed by Tukey's honest significant difference (HSD) test using Prism 5.0. Differences with a *p*‐value ≤0.05 were considered significant. Data are expressed as mean ± SEM from at least three independent experiments.

## 3 RESULTS

### 3.1 Expression and localization of GATAD2B during pre‐implantation embryonic development

**FIGURE 1:**
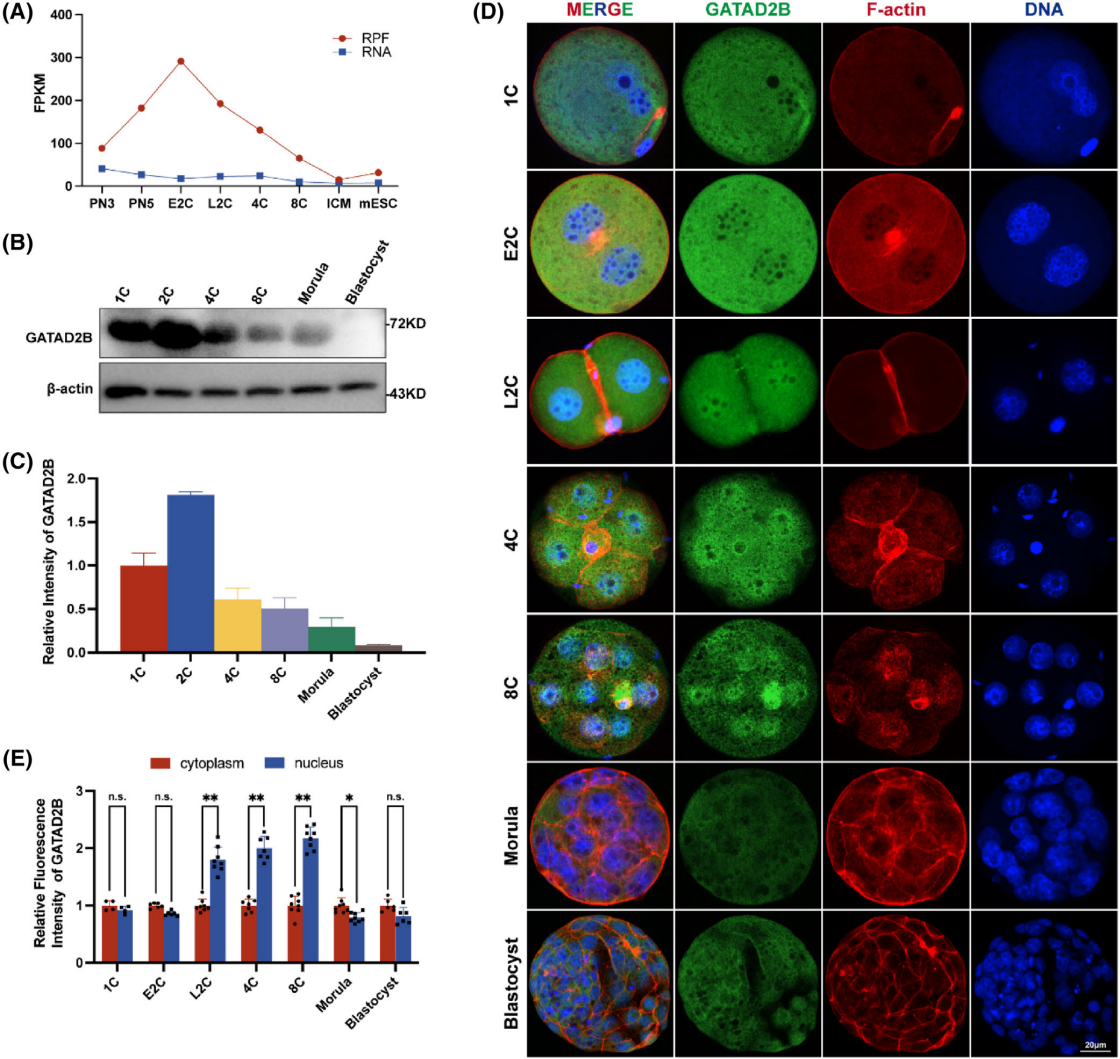
Expression and localization of GATAD2B during pre‐implantation early embryonic development. (A) Dynamic changes in ribosome‐associated RNA expression (RPF) of *Gatad2b* mRNA from the zygote stage to the inner cell mass (ICM) stage in mice. The RPF line graph represents changes in RNA molecules bound to ribosomes using low‐input Ribo‐seq (Ribo‐lite). The RNA line graph represents mRNA sequencing (mRNA‐seq). RPF refers to ribosome‐protected fragment. Other abbreviations include PN3 (early one‐cell stage), PN5 (late one‐cell stage), E2C (early 2‐cell stage), L2C (late 2‐cell stage), 4C (4‐cell stage), 8C (8‐cell stage), and ICM (inner cell mass). (B) Western blot analysis depicting the dynamic expression of GATAD2B protein level from the zygote stage to the blastocyst stage in mice. (C) The relative greyscale value histogram depicting the expression of GATAD2B at various stages of early embryonic development. (D) Immunofluorescence showing the localization of GATAD2B during pre‐implantation embryonic development (from zygote stage to blastocyst stage). GATAD2B is labelled in green. F‐actin is labelled in red. DAPI is labelled in blue. Scale bar, 20 μm. (E) Relative fluorescence expression histogram depicting the nuclear and cytoplasmic distribution of GATAD2B during early embryonic development. **p < 0.01. *p < 0.05. n.s., no significance.

Our previous studies revealed that GATAD2B is a maternally expressed factor predominant in oocytes, involved in regulating oocyte meiosis (unpublished data, under submission). Furthermore, we systematically compared the expression patterns of GATAD2B and other key members of the NuRD complex during early embryonic development (Figure S1). To preliminarily describe the dynamic changes of GATAD2B during pre‐implantation embryonic development, we reanalysed the changes of *Gatad2b* mRNA during mammalian oocyte‐to‐embryo transition and pre‐implantation development.^19^ *Gatad2b* mRNA expression remained stable at various stages of pre‐implantation embryonic development, but ribosome profiling (low‐input Ribo‐seq) revealed a significant increase during the early 2‐cell stage, followed by a rapid decrease in *Gatad2b* ribo‐binding mRNA from the late 2‐cell stage to the blastocyst stage (Figure 1A). The expression pattern of GATAD2B was further validated by protein Western blot analysis (Figure 1B,C). Interestingly, we found that during pre‐implantation embryonic development, GATAD2B exhibited specific nuclear enrichment from the late 2‐cell stage to the 8‐cell stage, while it did not localize to the nucleus during the morula to blastocyst stages (Figure 1D,E). These results not only suggest a potential role for GATAD2B during the late 2‐cell stage to the 8‐cell stage, but also indicate its possible involvement in transcriptional regulation due to its specific nuclear localization.

### 3.2 Knockdown of GATAD2B leads to abnormal blastocyst development

**FIGURE 2:**
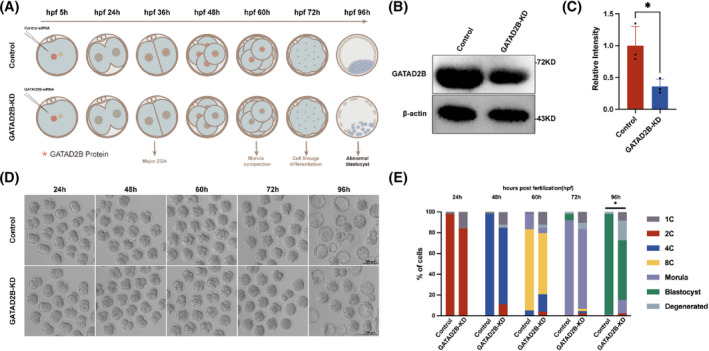
GATAD2B knockdown leads to abnormal embryonic development. (A) Experimental design diagram for GATAD2B knockdown. Briefly, GATAD2B siRNA pool was injected at the zygote stage to achieve GATAD2B knockdown, followed by monitoring the developmental progress of early embryos at different stages. (B) Western blot experiment showing the reduced protein levels of GATAD2B due to knockdown. (C) The bar graph quantitatively illustrates the knockdown efficiency of GATAD2B. **p < 0.01. *p < 0.05. n.s., no significance. (D) Representative images of GATAD2B‐KD and control embryos at different developmental stages. Scale bar, 100 μm. (E) Bar graph representing the proportions of embryos at different developmental stages. hpf, hours post‐fertilization.

To further investigate the role of GATAD2B during pre‐implantation embryonic development, we performed knockdown experiments targeting GATAD2B. Considering the increased protein expression of GATAD2B during the transition from the one‐cell stage to the two‐cell stage, we attempted knockdown experiments targeting *Gatad2b* mRNA starting from the one‐cell stage (Figure 2A–C). We found that the proportion of four‐cell embryos in the GATAD2B‐KD group began to decrease (control 98% vs. GATAD2B‐KD 70%). In the blastocyst stage, the rate of blastocyst formation in the GATAD2B‐KD group showed a significant decrease, with only 50% of embryos reaching the blastocyst stage, while the control group had a blastocyst rate higher than 90% (Figure 2D,E).

**FIGURE 3:**
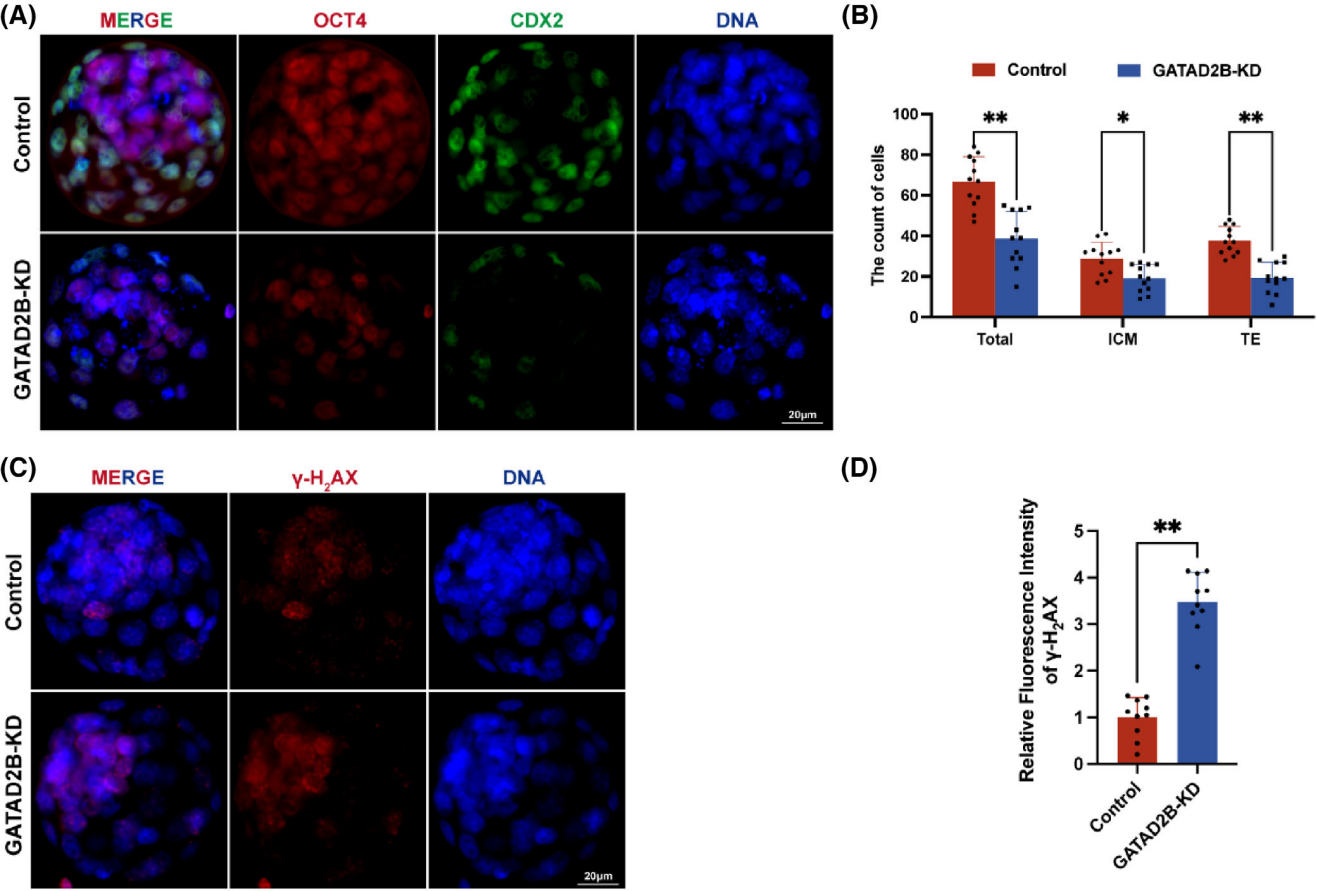
GATAD2B knockdown affects blastocyst lineage differentiation. (A) Immunofluorescence staining showing blastocyst lineage differentiation. OCT4, labelled in red, marks the inner cell mass (ICM). CDX2, labelled in green, marks the trophectoderm (TE). Scale bar, 20 μm. (B) Bar graph showing the quantification of the two cell types (ICM and TE) in control and GATAD2B‐KD blastocysts. (C) Immunofluorescence showing DNA damage in blastocysts. DNA damage is marked by γ‐H2AX. Scale bar, 20 μm. (D) Bar graph showing the relative fluorescence intensity of γ‐H2AX expression in blastocysts. **p < 0.01. *p < 0.05. n.s., no significance.

To further assess whether there were abnormalities in lineage differentiation within the blastocysts, we labelled the inner cell mass (ICM) and trophectoderm (TE) using OCT4 and CDX2 markers, respectively. We found that in GATAD2B‐KD blastocysts, the number of cells in the ICM and TE significantly decreased, and the total cell number in the blastocyst stage also significantly decreased (Figure 3A,B). To evaluate the differences in blastocyst quality, we used γ‐H2AX staining to assess the extent of DNA damage in blastocysts. We found a significant increase in DNA damage in the cells of GATAD2B‐KD blastocysts (Figure 3C,D). These results indicate that knockdown of GATAD2B significantly reduces blastocyst development rate and leads to decreased blastocyst quality.

### 3.3 Knockdown of GATAD2B leads to abnormal development of morula embryos

**FIGURE 4:**
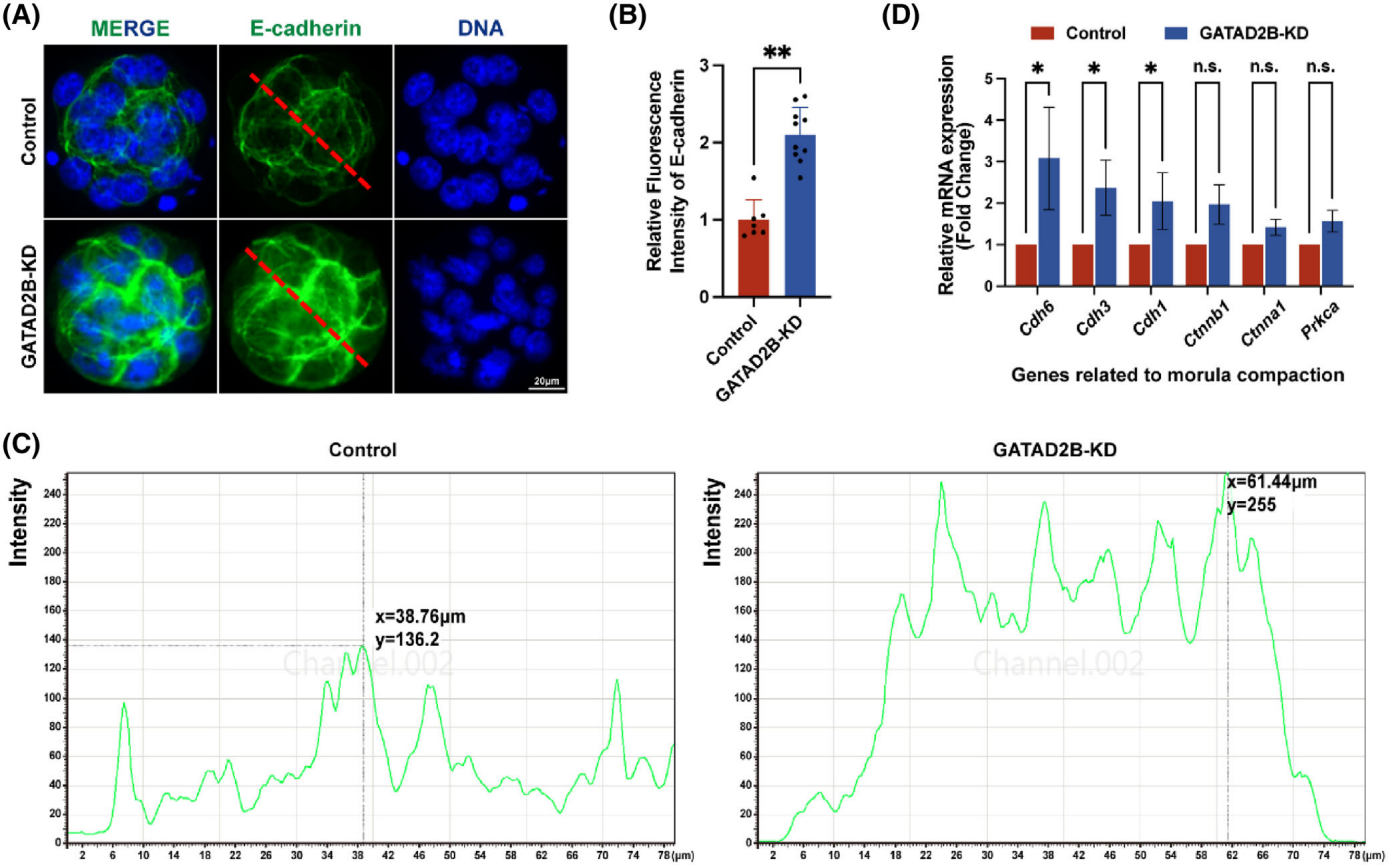
GATAD2B knockdown affects compaction of morula. (A) Immunofluorescence staining assessing morula development. E‐cadherin, labelled in green, represents the marker for morula compaction. Scale bar, 20 μm. (B) Bar graph depicting the relative fluorescence intensity of E‐cadherin expression in morula. (C) The greyscale values of E‐cadherin in Morula embryos are shown in the longitudinal section for both the control and GATAD2B‐KD groups, as depicted in the diagram. (D) qRT‐PCR showing the expression of mRNA related to morula compaction. **p < 0.01. *p < 0.05. n.s., no significance.

Although there was no significant difference in the number of morula embryos between the control group and the GATAD2B‐KD group, the details of morula embryonic development were observed to further evaluate the cause of abnormal development. Embryo compaction is a crucial event in morula embryonic development, and we carefully evaluated the molecules involved in the process of morula compaction. E‐cadherin, a key protein‐mediating cell–cell adhesion in mouse morula‐stage embryos,^20^ exhibited a slight increase in protein expression distribution, as demonstrated by immunofluorescence (Figure 4A,B). To provide a clearer description of the distribution of E‐cadherin, we displayed the corresponding greyscale values of E‐cadherin through a longitudinal section of the morula, as shown in Figure 4C. In GATAD2B‐KD morula, there was a significant enhancement in greyscale values at the cell junctions, with a comparison of Y‐axis greyscale values of 136.2 versus 255 (control vs. GATAD2B‐KD). Here, the *X*‐axis represents the distance from the morula's subcortical region. Consistent with the immunofluorescence results, qRT‐PCR analysis revealed varying degrees of upregulation in the expression of molecules involved in morula embryo compaction, including *Cdh6*, *Cdh3*, *Cdh1*, *Ctnnb1*, *Ctnna1*, and *Prkca* (Figure 4D). These results suggest that the loss of GATAD2B function leads to abnormal compaction involved in morula embryo.

### 3.4 Disruption of the transcriptome changes during the late 2‐cell embryo by GATAD2B functional loss

**FIGURE 5:**
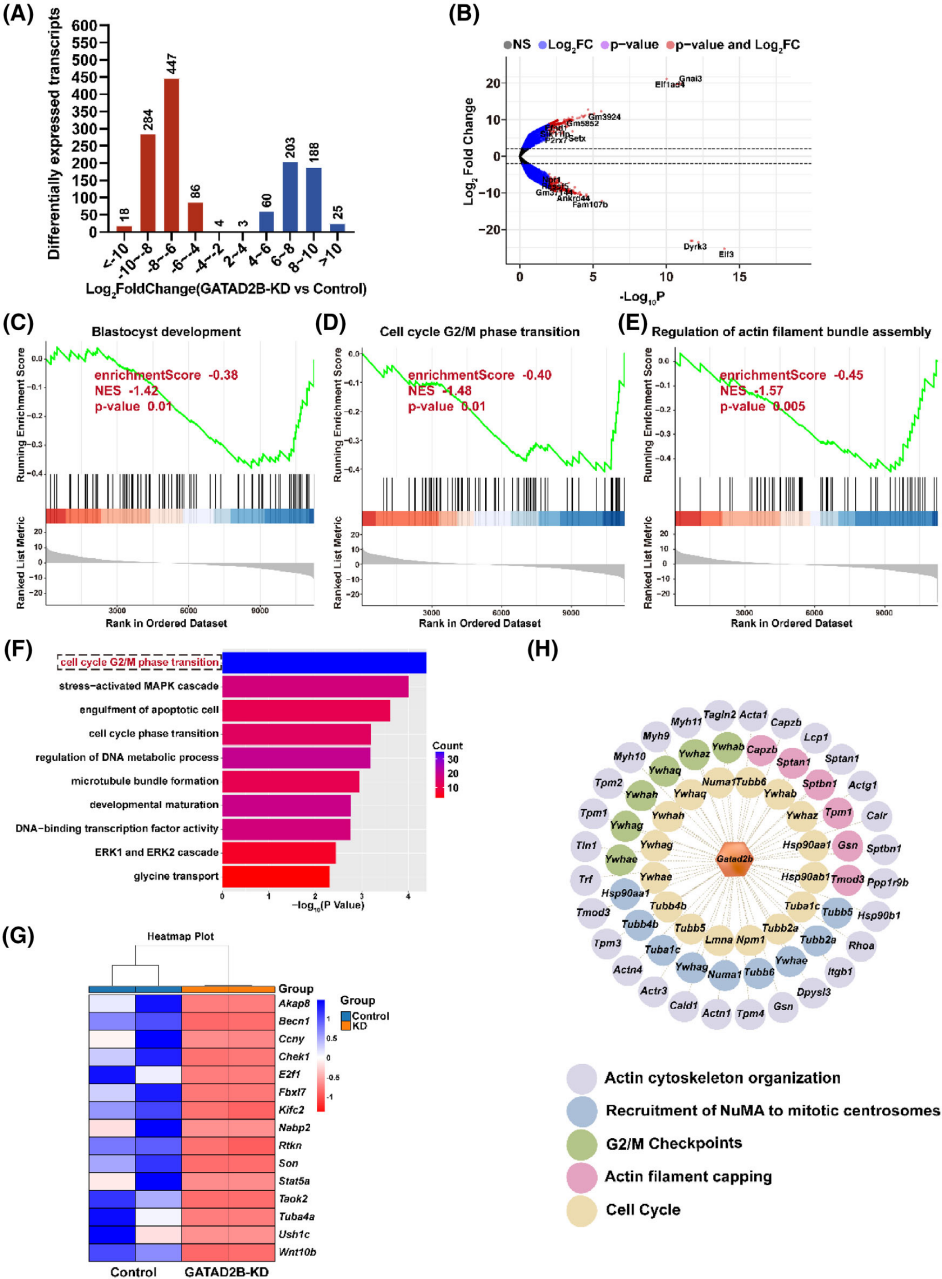
RNA‐seq and mass spectrometry analyses suggest the impact of GATAD2B deletion on cell cycle progression. (A) Distribution of differentially expressed transcripts at various levels between GATAD2B‐KD and control 2‐cell‐stage embryos, as detected by RNA‐seq. The number of changed transcripts is shown above each bar. (B) Volcano plot displaying the significant changes in transcripts identified by RNA‐seq analysis in GATAD2B‐KD 2‐cell‐stage embryos. Representative genes with significant differential expression are labelled in the plot. (C) Gene Set Enrichment Analysis (GSEA) results showing the suppression of the ‘blastocyst development’ signalling pathway in GATAD2B‐KD 2‐cell‐stage embryos. (D) GSEA results showing the suppression of the ‘cell cycle G2/M phase transition’ signalling pathway in GATAD2B‐KD 2‐cell‐stage embryos. (E) GSEA results showing the suppression of the ‘regulation of actin filament bundle assembly’ signalling pathway in GATAD2B‐KD 2‐cell‐stage embryos. (F) Bar chart illustrating the enrichment of Gene Ontology (GO) terms associated with significantly different transcripts in GATAD2B‐KD 2‐cell‐stage embryos. (G) Gene list of differentially expressed genes related to ‘cell cycle G2/M phase transition’. (H) Characterization of GATAD2B‐bound proteins identified by immunoprecipitation–mass spectrometry (IP‐MS).

To further investigate the reasons for the impact of GATAD2B knockdown on early embryonic development, we performed transcriptome sequencing during the late 2‐cell embryo stage. Transcriptome data revealed significant differences in the transcriptomes of GATAD2B‐KD embryos. Among them, 839 transcripts were significantly downregulated, and 479 transcripts were significantly upregulated in the GATAD2B‐KD group (fold change>4). The number of downregulated transcripts was 1.75 times higher than the number of upregulated transcripts (Figure 5A,B). Gene Set Enrichment Analysis (GSEA) captured several gene sets with differential expression in the transcriptome changes. GSEA indicated significant downregulation of the ‘blastocyst development’ signalling pathway associated with blastocyst development, the ‘regulation of actin filament bundle assembly’ pathway associated with morula embryo compaction, and the ‘cell cycle G2/M phase transition’ pathway associated with embryo cleavage (Figure 5C–E). We further conducted GO pathway enrichment analysis for the 839 downregulated transcripts in GATAD2B‐KD late 2‐cell embryos. These downregulated transcripts were enriched in cell cycle‐related signalling pathways, including ‘cell cycle G2/M phase transition’, ‘cell cycle phase transition’, and ‘development maturation’ (Figure 5F). Based on the earlier observation that the proportion of four‐cell embryos and eight‐cell embryos at the same developmental time was lower in the GATAD2B‐KD group compared to the control group, we further analysed the ‘cell cycle G2/M phase transition’ signalling pathway. We found significant downregulation of important factors regulating cell cycle transition, such as *Akap8*, *Becn1*, *Ccny*, and *Chek1* (Figure 5G). To further elucidate the mechanisms underlying the impact of GATAD2B on cell cycle transitions, we conducted additional data mining of our previous ovarian proteomic data. Interestingly, we discovered that proteins interacting with GATAD2B also converged on signalling pathways related to ‘cell cycle’ and ‘G2/M phase transition’ (Figure 5H). These results suggest that GATAD2B knockdown affects the dynamic changes in the transcriptome during the late 2‐cell embryo stage, particularly highlighting the inhibition of cell cycle‐related signalling pathways.

### 3.5 Knockdown of GATAD2B disrupts histone acetylation processes

**FIGURE 6:**
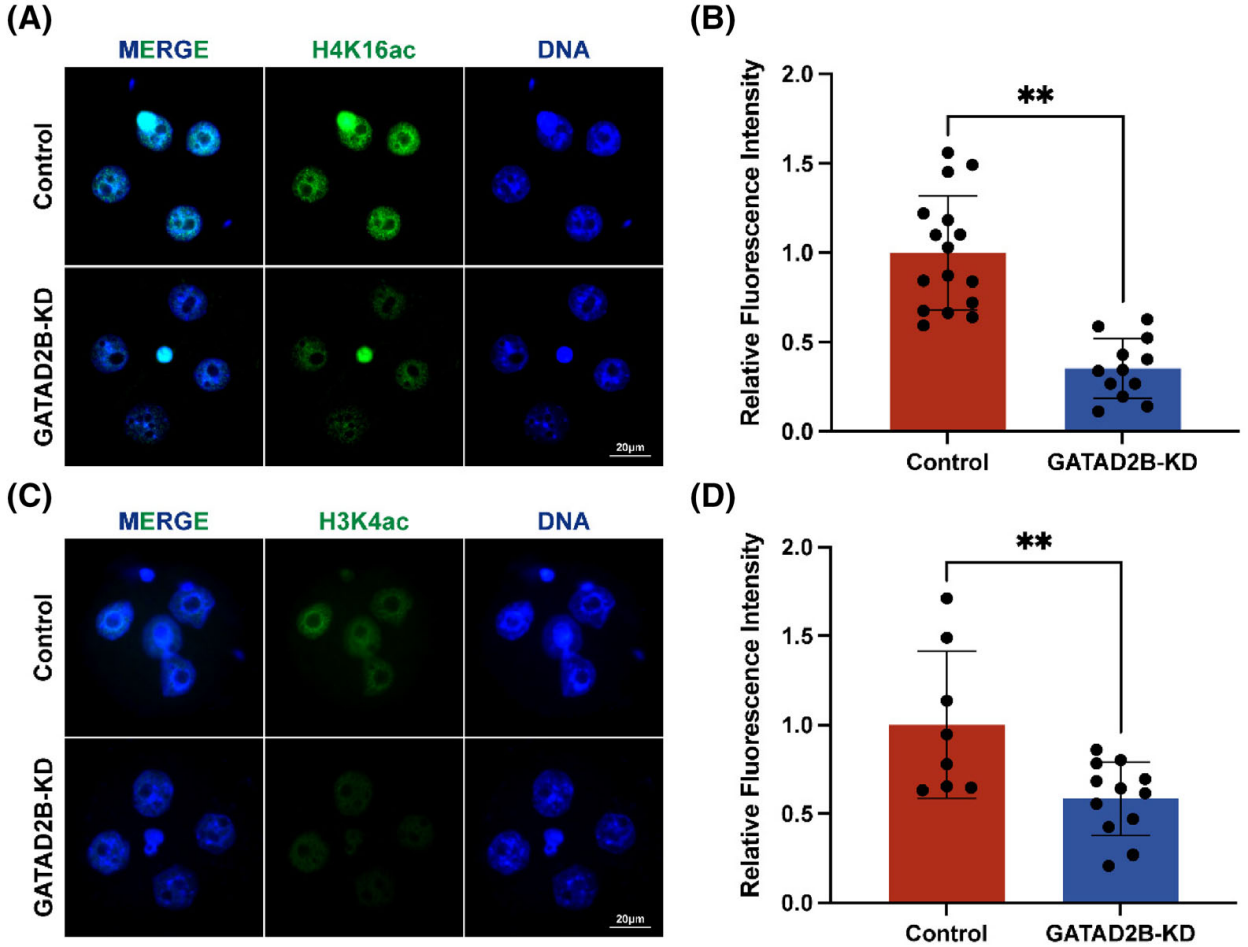
GATAD2B knockdown disrupts histone acetylation processes. (A) H4K16ac staining in control and GATAD2B‐KD 4C embryos. Scale bar, 20 μm. (B) Relative fluorescence intensity of H4K16ac staining in control and GATAD2B‐KD 4C embryos. (C) H3K4ac staining in control and GATAD2B‐KD 4C embryos. Scale bar, 20 μm. (D) Relative fluorescence intensity of H3K4ac staining in control and GATAD2B‐KD 4C embryos.

As GATAD2B is a core component of the nucleosome remodelling and histone deacetylation (NuRD) complex and is closely associated with histone acetylation and transcriptional activation,^9^ we investigated the changes in histone acetylation upon GATAD2B knockdown. Previous studies have reported that maternal deletion of H4K16ac leads to abnormal activation of ZGA.^10^ In our study, we found a significant decrease in the expression of H4K16ac in GATAD2B knockdown embryos (Figure 6A,B). Furthermore, it is known that H3K4ac plays a positive role in transcription.^21^ Consistently, immunofluorescence analysis revealed a significant downregulation of H3K4ac expression in GATAD2B‐deficient embryos (Figure 6C,D). These results clearly indicate that knockdown of GATAD2B disrupts the histone acetylation processes during pre‐implantation early embryonic development.

### 3.6 Loss of GATAD2B function impairs zygotic genome activation

**FIGURE 7:**
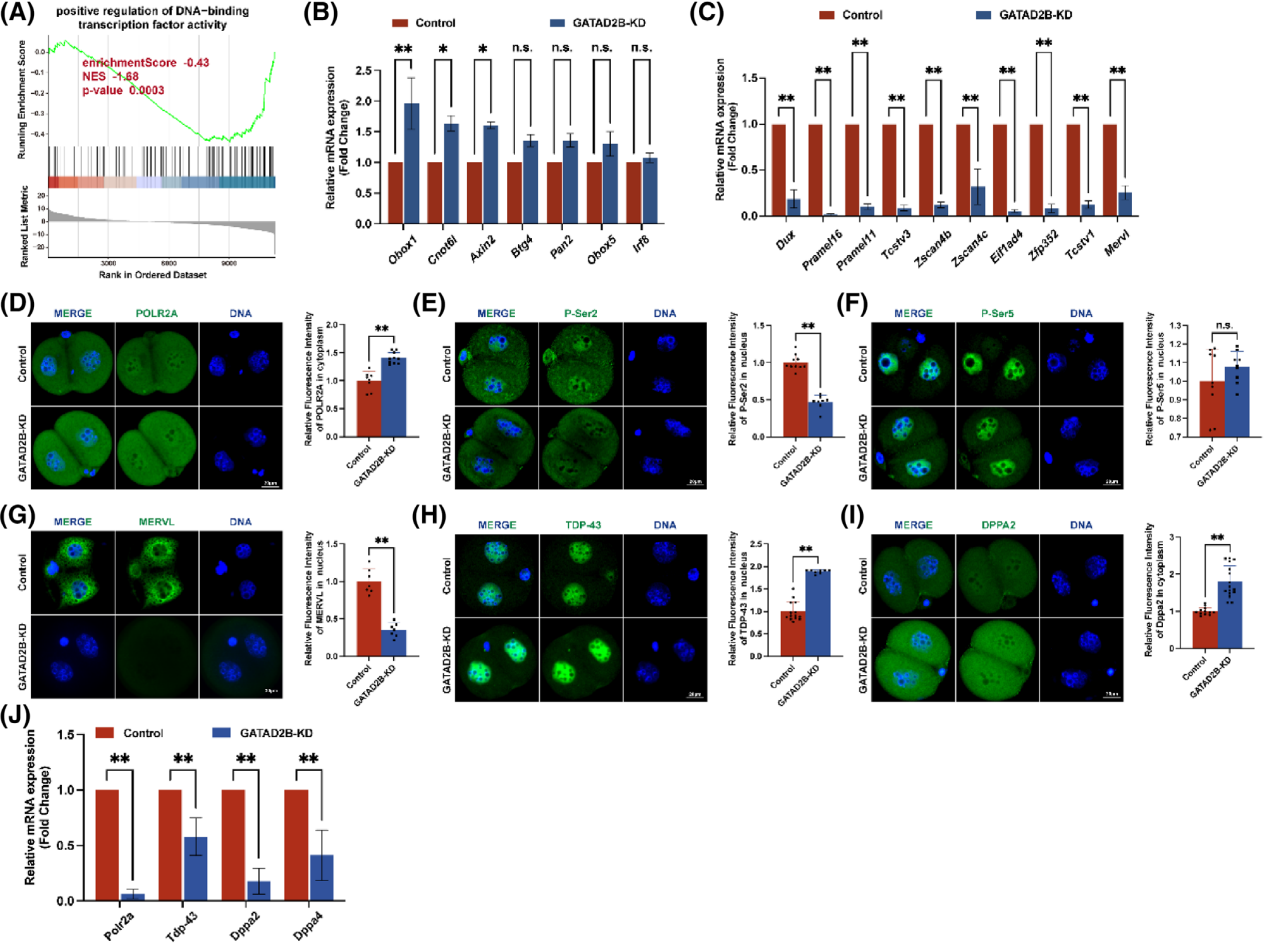
Loss of GATAD2B function impairs zygotic genome activation. (A) GSEA results showing the suppression of the ‘positive regulation of DNA‐binding transcription factor activity’ signalling pathway in GATAD2B‐KD 2‐cell‐stage embryos. (B) qRT‐PCR demonstrating changes in maternal mRNA at the 2‐cell stage. (C) qRT‐PCR demonstrating changes in ZGA‐related gene mRNA at the L2C embryos. (D) POLR2A staining in control and GATAD2B‐KD L2C embryos. Scale bar, 20 μm. (E) P‐Ser2 staining in control and GATAD2B‐KD L2C embryos. Scale bar, 20 μm. (F) P‐Ser5 staining in control and GATAD2B‐KD L2C embryos. Scale bar, 20 μm. (G) MERVL staining in control and GATAD2B‐KD L2C embryos. Scale bar, 20 μm. (H) TDP‐43 staining in control and GATAD2B‐KD L2C embryos. Scale bar, 20 μm. (I) DPPA2 staining in control and GATAD2B‐KD L2C embryos. Scale bar, 20 μm. (J) qRT‐PCR demonstrating changes in several gene mRNA at the L2C embryos. **p < 0.01, *p < 0.05. n.s., no significance.

The unique nuclear localization of GATAD2B at the late two‐cell, four‐cell, and eight‐cell embryos prompted us to investigate its relationship with zygotic genome activation. Consistent with GATAD2B's potential involvement in transcriptional regulation, GSEA of the GATAD2B‐KD transcriptome indicated a significant downregulation of ‘DNA‐binding transcription activity’ (Figure 7A). During the transition from oocyte fertilization to early embryonic development, extensive degradation of maternal mRNAs occurs. Simultaneously, the embryonic genome at the two‐cell stage initiates gene transcription and synthesizes new RNAs, a process known as embryonic genome activation.^4^, ^22^ We found that in GATAD2B‐KD two‐cell embryos, the degradation of maternal transcripts such as *Obox1*, *Cnot6l*, and *Axin2*, was slowed down. Additionally, key genes expressed at the two‐cell stage, such as *Dux*, *Zscan4b*, *Zscan4c*, and *Tcstv1*, as well as critical transposable elements of the two‐cell embryonic genome, including MERVL, showed lower expression levels compared to the control group (Figure 7B,C).

The activity of the transcription machinery RNA Pol II is necessary for successful major ZGA and embryonic development.^5^ To investigate this, we performed staining for the largest subunit of RNA Pol II, POLR2A, in control and GATAD2B‐KD late 2‐cell embryos. Compared to the control group, the signal of POLR2A staining in the cytoplasm showed a significant increase (Figure 7D). Phosphorylation of the C‐terminal domain (CTD) of POLR2A is crucial for regulating transcriptional timing, with phosphorylation at Ser2 (P‐Ser2) or Ser5 (P‐Ser5) of the CTD directly involved in modulating RNA Pol II activity.^23^, ^24^ In GATAD2B‐KD late 2‐cell embryos, the signal of P‐Ser2 in the nucleus was significantly reduced, while no difference was observed in the signal of P‐Ser5 in the nucleus (Figure 7E,F). MERVL (murine endogenous retrovirus‐L), a marker of totipotency, exhibits rapid and high expression during the two‐cell embryo stage. Depletion of MERVL transcripts leads to abnormal lineage differentiation and destabilization of the genomic integrity, resulting in developmental disorders.^25^ Since the transcripts of MERVL were significantly reduced in GATAD2B‐deficient two‐cell embryos, we further evaluated the differential changes of MERVL at the two‐cell and four‐cell stages. In the absence of GATAD2B, MERVL expression was lower in two‐cell embryos (Figure 7G).

In addition, recent studies have identified TDP‐43, DPPA2/4, and others as important regulators of ZGA proteins. TDP‐43 and DPPA2/4 play significant roles in mouse early embryonic development by influencing RNA Pol II configuration and zygotic genome activation.^6^, ^26^ Compared to the control group embryos, GATAD2B‐KD embryos exhibited abnormal increases in nuclear TDP‐43 signal, while the signal of DPPA2 showed a significant increase only in the cytoplasm (Figure 7H,I). These results suggest that GATAD2B knockdown leads to impaired genome activation in late 2‐cell embryos, resulting in compensatory increases in TDP‐43 and DPPA2 expression. Consistent with these findings, it is known that TDP‐43 and DPPA2/4 are maternal mRNAs.^6^, ^26^ When these maternal proteins are translationally upregulated in the late 2‐cell stage, the corresponding transcripts show a significant decrease (Figure 7J).

Collectively, these results indicate that the loss of GATAD2B affects the process of zygotic genome activation.

## 4 DISCUSSION

GATAD2B begins to exhibit distinct nuclear localization at the late two‐cell stage but no longer localizes to the nucleus at the morula stage, which piqued our interest. First, we analysed the protein structure of GATAD2B, which contains a typical GATA‐type zinc finger (Znf), CR1 region, and CR2 region. The presence of GATA‐type zinc fingers indicates that GATAD2B is a GATA‐binding transcription factor, and the highly conserved Znf domains coordinate the zinc ion through four cysteine residues.^27^ Znf domains can specifically bind to the DNA sequence (A/T)GATA(A/G)^28^ in the regulatory regions of genes. Indeed, we observed a significant downregulation of ‘DNA‐binding transcription activity’ in GATAD2B‐depleted two‐cell embryos. The NuRD complex, composed of several core subunits, including *Hdac1*/*2*, *Mta1*/*2*/*3*, *Rbbp4*/*7*, *Chd3*/*4*, and *Gatad2a*/*2b*, regulates transcription through nucleosome remodelling and histone deacetylation.^18^, ^29^ However, among these core subunits, *Chd3*, *Mta1*, and *Hdac2* are either lowly expressed or not expressed at the two‐cell stage. *Rbbp7* is not expressed in the late two‐cell and four‐cell stages, while *Gatad2b* and *Chd4* exhibit the highest expression in the early two‐cell stage.^19^ These findings suggest that the core subunits of the NuRD complex have different expression patterns during early embryonic development, indicating potential functional differences. Similar to *Gatad2b*, *Chd4* and *Gatad2a* may play a role in the process of embryonic genome activation during the 2‐cell stage. However, due to the low expression of *Chd3*, *Mta1*, and *Hdac2* in pre‐implantation early embryonic development, they may not have a functional role in this process. Additionally, *Mta3* and *Rbbp4*, which are highly expressed at the 8‐cell stage, may have specific functions in the compaction process of the embryo. The specific functions of these core subunits of the NuRD complex still warrant further exploration.

To further explore the structural basis of GATAD2B nuclear localization, we examined two distinctive structural domains of GATAD2B: CR1 and CR2 regions. It is known that both CR1 and CR2 regions are required for speckled nuclear localization,^14^, ^30^, ^31^ providing a structural basis for GATAD2B. In somatic cells, CR1 interacts with MBD2 and MBD3, enhancing MBD2‐mediated repression.^14^, ^30^ Additionally, GATAD2B targets MBD3 to discrete loci in the nucleus.^31^ The conserved CR2 region of GATAD2B interacts with the tails of all octamer histones in vitro, and acetylation of histone tails interferes with p66 binding.^14^ Although it is challenging to obtain early embryonic tissues, our previous ovarian proteomic data supported the binding of GATAD2B to various types of histones (Figure S2). However, further investigation is needed to elucidate how GATAD2B regulates these changes in histone binding.

The zygotic genome activation (ZGA) is the initial transcription event that is crucial for embryonic development. Minor ZGA occurs at the middle 1‐cell (1C) stage, while major ZGA takes place at the late 2‐cell (L2C) stage in mice.^4^ Whether GATAD2B is involved in regulating ZGA is an area of interest for us. GATAD2B localizes to the nucleus from the late 2‐cell, 4‐cell, and 8‐cell stages. This period coincides with the timing of ZGA. When GATAD2B is depleted, it not only delays the progression from the two‐cell stage to the four‐cell stage but also leads to abnormal lineage differentiation of the blastocyst. The phenotype may be influenced by the varying knockdown efficiency of GATAD2B protein. In addition, we aimed to provide evidence of changes in key marks associated with ZGA activation in GATAD2B‐depleted two‐cell embryos. Transcription factors of the DUX family are known to be key regulators of ZGA in placental mammals.^32^, ^33^ The high expression of the MERVL long terminal repeat (LTR) promoter during major ZGA drives a subset of two‐cell genes and generates chimeric transcripts with host genes.^34^ When GATAD2B is absent during the two‐cell stage, the timely activation of key regulatory elements, such as DUX and the transposon MERVL, is impaired. GATAD2B may be an upstream regulatory molecule of MERVL, but whether it directly or indirectly influences MERVL requires further investigation.

In addition, a previous study has reported that during the transition from mouse oocytes to embryos, the transcription machinery of RNA Pol II undergoes loading, pre‐configuration, and elongation for gene expression.^5^ The coordination of transcription events relies on the phosphorylation of the C‐terminal domain (CTD) of POLR2A, particularly the phosphorylation of Ser2 (P‐Ser2) or Ser5 (P‐Ser5) of CTD, which can regulate the initiation and elongation of RNA Pol II transcription.^23^, ^24^ During the late 2‐cell stage, the loss of GATAD2B leads to the downregulation of Ser2 (P‐Ser2) on CTD, but its effect on Ser5 (P‐Ser5) remains unclear. This detail has caught our attention, although the underlying reasons are yet to be determined. Recently, several factors, such as DUX, DPPA2/4, TDP‐43, and KLF17, have been identified as important regulators of zygotic genome activation (ZGA) genes.^6^, ^26^, ^35^ Among them, DPPA2/4 binding to the Dux promoter leads to upregulation of Dux and activation of the 2C‐like transcriptional programme in ‘2C‐like’ cells.^26^ Maternal TDP‐43 is critical for mouse early embryonic development by affecting RNA Pol II configuration and zygotic genome activation.^6^ Interestingly, during the late 2‐cell stage, the loss of GATAD2B results in a significant increase in TDP‐43 and DPPA2, which may be a compensatory response due to the hindered RNA Pol II configuration. While our findings clearly indicate that the loss of GATAD2B impairs RNA Pol II pre‐configuration and causes changes in the expression of ZGA‐related proteins, such as TDP‐43 and DPPA2, the interaction between GATAD2B and these ZGA‐related proteins warrants further investigation.

When GATAD2B is knocked down, it leads to various abnormal phenotypes in embryonic development, and we were particularly interested in the changes of E‐cadherin during the morula stage. In a physiological state, E‐cadherin localizes on all cell surfaces during the 8‐cell stage and becomes restricted to cell contacts in the morula stage. The localization of E‐cadherin is a dynamic process in the 8‐cell stage, morula, and blastocyst.^36^ We observed an excessive enrichment of E‐cadherin at cell contacts in the GATAD2B‐KD group's morula. If E‐cadherin is excessively distributed at cell contacts, it can affect cell polarity.^37^ Mutant morulae devoid of E‐cadherin fail to compact and exhibit ectopic aPKC membrane staining.^37^ Furthermore, the expression of the cell–cell adhesion molecule E‐cadherin is involved in regulating the normal lineage segregation of inner and outer cells, particularly the regulation of *Cdx2* expression.^38^ These discussions indicate that the expression and localization of E‐cadherin need to be within a certain range. When E‐cadherin expression is too high, it may affect lineage differentiation, thereby leading to a decrease in the quality of the blastocyst. However, the direct causes of this phenomenon still require further investigation in subsequent studies.

In summary, our study elucidates the dynamic changes of GATAD2B during early embryonic development, where it exhibits a unique nuclear localization solely during the late 2‐cell stage to the 8‐cell stage. Depletion of GATAD2B affects early embryonic development and leads to abnormalities in blastocyst lineage differentiation. Transcriptome and qRT‐PCR analyses suggest that the embryonic developmental abnormalities caused by GATAD2B knockdown may be due to aberrant activation of the zygotic genome (ZGA). Our findings provide insights into the understanding of pre‐implantation embryonic developmental defects.

## AUTHOR CONTRIBUTIONS

GY‐C, HX‐S and GJ‐Y contributed to the conception and design of the study. YL‐L, LN‐Y, Q‐X, PP‐Q, Y‐Z, XH‐D and GY‐C performed the research. YL‐L and GY‐C performed the statistical analysis. GY‐C wrote the first draft of the manuscript. All authors contributed to manuscript revision and read and approved the submitted version.

## FUNDING INFORMATION

This work was supported by the National Natural Science Foundation of China (32000563), State Key Laboratory of Reproductive Medicine and Offspring Health (SKLRM‐2022D2), Clinical Trials from the Affiliated Drum Tower Hospital, Medical School of Nanjing University (2023‐LCYJ‐PY‐35), Startup Funds from Nanjing Drum Tower Hospital (RC2022‐019), and Key Laboratory of Reproductive Medicine of Guangdong Province (2020B1212060029).

## CONFLICT OF INTEREST STATEMENT

The authors declare no conflict of interest.

## Supporting information


**Figure S1.** Dynamic Changes of Major Components of the NuRD Complex from Zygote Stage to Blastocyst Stage. (A) Schematic description of the NuRD complex. (B) Dynamic changes in ribosome‐associated RNA expression (RPF) of NuRD complex components from the zygote stage to the inner cell mass (ICM) stage in mice. The RPF line graph represents changes in RNA molecules bound to ribosomes using low‐input Ribo‐seq (Ribo‐lite). The RNA line graph represents conventional mRNA sequencing (mRNA‐seq). RPF refers to ribosome‐protected fragments. Other abbreviations include PN3 (early one‐cell stage), PN5 (late one‐cell stage), E2C (early 2‐cell stage), L2C (late 2‐cell stage), 4C (4‐cell stage), 8C (8‐cell stage), and ICM (inner cell mass). Based on the observed patterns, the member genes of the NuRD complex have been categorized into four distinct clusters.

**Figure S2.** Protein Enrichment Analysis of GATAD2B‐Bound Proteins Identified by Mass Spectrometry, Related to ‘Histone Assembly’ and ‘DNA Transcription Genes’ (A) Network illustration of GATAD2B‐bound proteins that are crucial for transcriptional regulation. (B) Partial list of proteins obtained from GATAD2B ovarian mass spectrometry.

**Table S1.** Antibody list used in this study.

**Table S2.** Primer list used in this study.

**Table S3.** Protein list obtained from GATAD2B ovarian mass spectrometry.

## Data Availability

The data that supports the findings of this study are available in the supplementary material of this article.
