# Supplementary figures and images for: GATAD2B is required for pre‐implantation embryonic development by regulating zygotic genome activation
Source: Cell Prolif. 2024 Apr 12;57(9):e13647. doi: 10.1111/cpr.13647 (PMC11503246; doi:10.1111/cpr.13647)

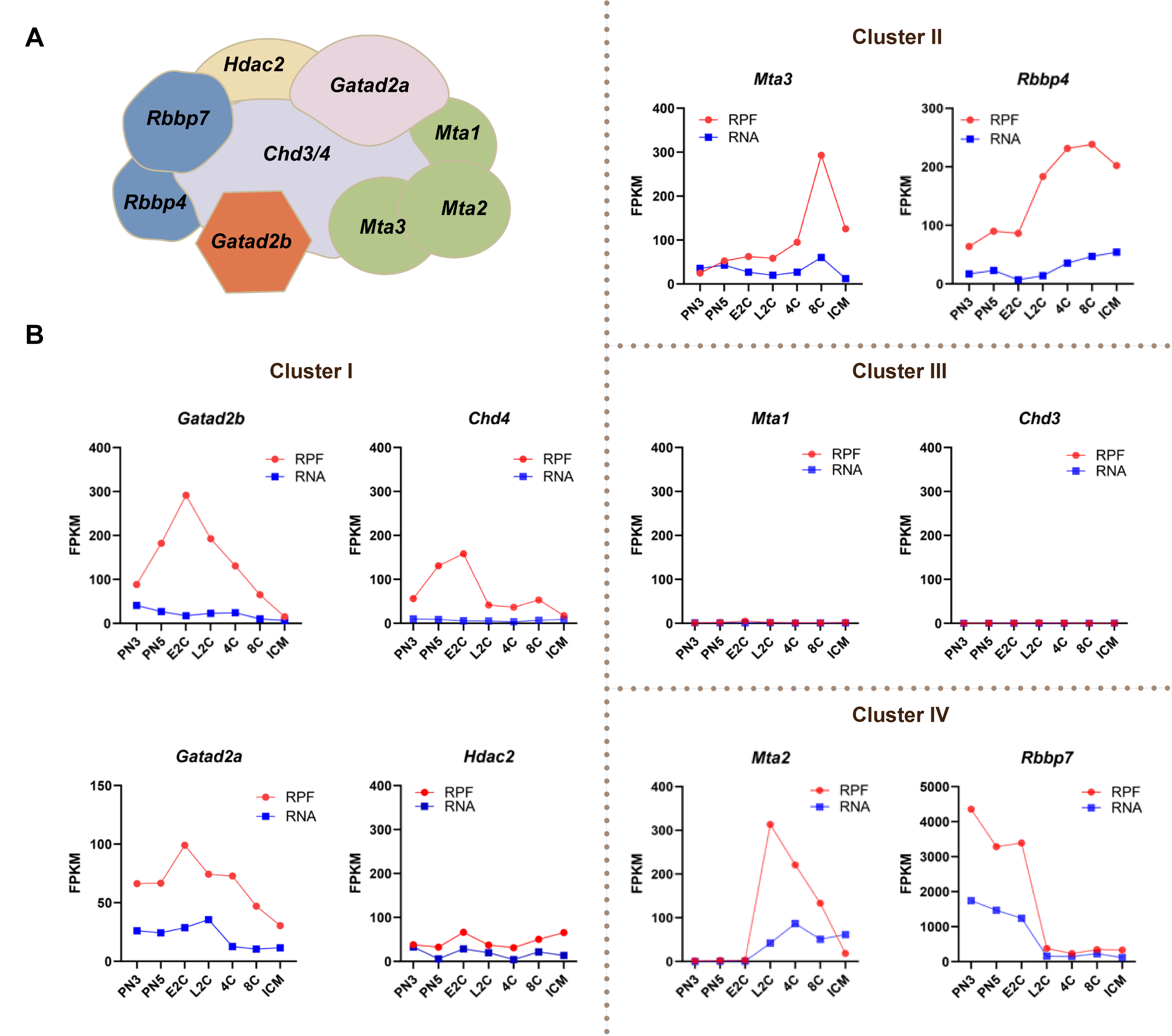

Supplement: Supplementary file 1 — Figure S1. Dynamic Changes of Major Components of the NuRD Complex from Zygote Stage to Blastocyst Stage. (A) Schematic description of the NuRD complex. (B) Dynamic changes in ribosome‐associated RNA expression (RPF) of NuRD complex components from the zygote stage to the inner cell mass (ICM) stage in mice. The RPF line graph represents changes in RNA molecules bound to ribosomes using low‐input Ribo‐seq (Ribo‐lite). The RNA line graph represents conventional mRNA sequencing (mRNA‐seq). RPF refers to ribosome‐protected fragments. Other abbreviations include PN3 (early one‐cell stage), PN5 (late one‐cell stage), E2C (early 2‐cell stage), L2C (late 2‐cell stage), 4C (4‐cell stage), 8C (8‐cell stage), and ICM (inner cell mass). Based on the observed patterns, the member genes of the NuRD complex have been categorized into four distinct clusters. [file CPR-57-e13647-s002.tif]

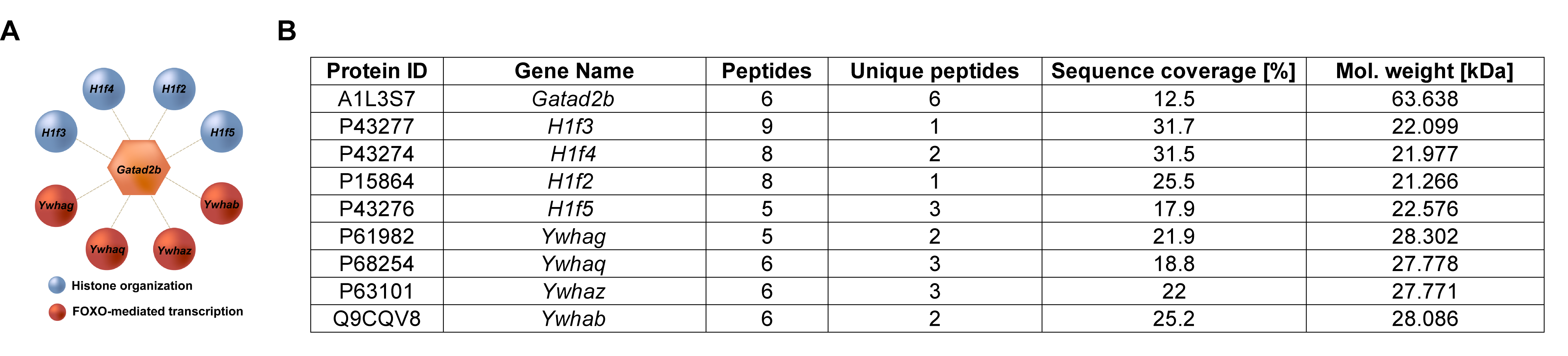

Supplement: Supplementary file 2 — Figure S2. Protein Enrichment Analysis of GATAD2B‐Bound Proteins Identified by Mass Spectrometry, Related to ‘Histone Assembly’ and ‘DNA Transcription Genes’ (A) Network illustration of GATAD2B‐bound proteins that are crucial for transcriptional regulation. (B) Partial list of proteins obtained from GATAD2B ovarian mass spectrometry. [file CPR-57-e13647-s005.tif]
